# A yellow fever 17D and Usutu virus chimera with rationally designed mutations in the envelope protein is lethal in an Ifnar^-/-^ mouse model

**DOI:** 10.1186/s12985-026-03145-x

**Published:** 2026-07-03

**Authors:** Johanna M. Duyvestyn, Peter J. Bredenbeek, Melissa Thaler, Marjolein Kikkert, Martijn J. van Hemert

**Affiliations:** Molecular Virology Laboratory, dept. Leiden University Center for Infectious Diseases (LUCID), Leiden University Medical Center, Leiden, The Netherlands

## Abstract

The expanding geographical spread of Usutu virus (USUV) poses an increasing threat to bird populations and to human health. Here we assessed a chimeric USUV vaccine candidate that was constructed using the live attenuated yellow fever virus (YFV) YF-17D platform. In our chimeric virus, the pre-membrane (PrM) and envelope (E) proteins of YFV were replaced with those of USUV. Similar chimeras of YF-17D with otherflaviviruses have been reported to be attenuated in vivo, making them promising modified live virus vaccine candidates. We used either wild type USUV PrME or USUV PrME containing rationally designed mutations in the E protein that were expected to (further) attenuate the virus, based on their effect in the context of other orthoflaviviruses. In cell culture the YF-17D/USUV chimeric viruses displayed reduced fitness compared to both YF-17D and USUV, with the chimera containing the mutated USUV E exhibiting the slowest growth kinetics. However, in our interferon α/β receptor deficient (Ifnar^−/−^) mouse model the chimeric YF-17D/USUV viruses caused rapid lethality in all animals, with survival times that were only marginally better compared to wild type USUV. It was surprising that introduction of the PrME region from the naturally low pathogenic USUV into the YF-17D platform yielded viruses that caused lethal infections in our model, while in contrast other neuroinvasive orthoflaviviruses are attenuated in this same chimeric platform. All control mice that were infected with a 1000-fold higher dose of YF-17D survived. We also discovered that the mutations in E that are attenuating in related viruses did not result in attenuation in the Ifnar^−/−^ mouse model, neither in the context of USUV itself or the YF-17D chimera. This may be due to the immunocompromised nature of the mice or because these mutations are not attenuating in the context of USUV. This work highlights some complications and unpredictability related to the use of the chimeric live attenuated YF-17D vaccine platform and introduction of rationally designed mutations with a predicted attenuating effect. Furthermore, our work illustrates that infection models that are able to better predict the phenotype and safety of (chimeric) USUV/orthoflavivirus vaccine candidates are needed.

**Supplementary Information:**

The online version contains supplementary material available at 10.1186/s12985-026-03145-x.

## Introduction

Orthoflaviviruses are a genus of arthropod-borne single stranded (+) RNA viruses, many of which can cause debilitating and potentially fatal disease and a high burden on global health [1]. The increase in epidemics of (re-)emerging orthoflaviviruses such as dengue virus (DENV), Japanese encephalitis virus (JEV), West Nile virus (WNV), and Zika virus (ZIKV) is being driven by socio-economic, ecological and environmental factors as well as unpredictable changes in the properties and pathogenicity of these viruses [2, 3]. Usutu virus (USUV) is an emerging mosquito-borne orthoflavivirus within the JEV serocomplex that has become endemic in an increasing number of countries since its spread out of Africa in recent decades [4]. Outbreaks can result in massive die offs in the passerine bird species that act as reservoir hosts in the enzootic transmission cycle, as well as spillover infections into numerous species of mammals. While infection in humans often results in mild or no symptoms, an increasing number of acute and neuroinvasive disease cases are being documented, with immunocompromised individuals more at risk [4, 5].

The 11 kb orthoflavivirus genome encodes 3 structural and 7 non-structural (NS) proteins. In the immature virion, the viral RNA and the Capsid (C) protein form a nucleocapsid structure, which is enveloped by a lipid bilayer containing the pre-membrane (prM) and envelope (E) proteins. Maturation of the virion requires proteolytic cleavage of prM by furin. The envelope protein contains the epitopes that are targeted by neutralizing immune responses, and it also determines cellular tropism by binding to a wide range of receptors. It thus plays an important role in pathogenicity of the virus [1, 6–8].

Due to the challenges in vector control, and the lack of antiviral therapeutics, vaccines are a vital tool in the fight against orthoflaviviruses [9]. Highly successful vaccines licensed for use in humans exist against yellow fever virus (YFV), JEV, DENV, and tick-borne encephalitis (TBEV), which consist of either live-attenuated virus (LAV) or inactivated viruses. Besides these more traditional vaccine designs, also many other novel and promising strategies are in development [3, 9–11]. Chimeric live-attenuated vaccine strategies - swapping the PrME region of a safe LAV with the PrME region from the virus of interest - are appealing for designing vaccines against emerging orthoflaviviruses. Such strategies can utilize well-characterised platforms, such as the YF-17D-based platform, which is highly immunogenic and has a robust safety profile [12, 13].

Though LAVs generally induce strong and long-lasting protection, they can have an increased safety risk compared to other vaccine platforms due to their replicative nature and the need for symptom-free clearance by the healthy immune system of the vaccinee. Therefore, safe-by-design strategies are increasingly employed to better control the risks of such vaccines [3, 11]. Specific considerations arise for chimeric flavivirus vaccine candidates because the determinants of virulence for neurotropic orthoflaviviruses map to the envelope protein [13]. Imojev, a YF-17D/JEV-PrME chimeric vaccine incorporates the PrME of the attenuated (serially passaged) variant JEV SA14-14-2 rather than from a wild type JEV [14, 15]. Furthermore, the mutations in the envelope protein implicated in attenuation of the JEV SA14-14-2 vaccine were assessed for their ability to improve the safety of a chimeric YF-17D/WNV-PrME vaccine candidate (ChimeriVax-WN). Three of these mutations were found to be stable and attenuating, thus were incorporated into the WNV envelope of the ChimeriVax-WN_02_ vaccine candidate. E-L107F, which showed the strongest attenuating effect in WNV, is a mutation in a highly conserved site of the fusion loop and is known to disrupt fusion peptide function in a broad range of orthoflaviviruses [6, 16–19]. E-A316V is located in the receptor binding domain and may disrupt cell entry. E-K440R is located in the transmembrane (TM) domain and may alter association with PrM [20]. The ChimeriVax-WN_02_ vaccine candidate containing these mutations showed reduced neurovirulence in both mice and non-human primates [20, 21].

USUV is closely related to JEV and WNV (71 and 68% nucleotide identity respectively across the entire genome), therefore we hypothesize that the attenuating substitutions that were conserved between JEV and WNV may have similar attenuating effects in USUV [22]. To test this, and to assess whether these mutations might also enhance the safety profile of an USUV vaccine candidate, we constructed a recombinant USUV containing these three mutations (L107F, A316V and K440R – referred to henceforth as FVR), which was characterised in vitro and in vivo. Two YF-17D chimeric viruses, incorporating either wild type USUV PrME or the mutated USUV PrME (containing the FVR substitutions) were also compared, both in cell lines and in preliminary survival studies in an Ifnar^−/−^ mouse model.

## Methods

### Viruses and cell lines

Usutu virus Africa-3 Lineage, isolate AS201600045 TM Netherlands 2016 (Af-3-NL), GenBank accession number MH891847 [23] was a kind gift from Erasmus Medical Center Rotterdam, The Netherlands. The working stock was passaged twice on Vero CCL-81 cells at 37 °C, 5% CO_2_ in Dulbecco’s modified Eagle’s medium (DMEM, Gibco) supplemented with 3% fetal calf serum (FCS, Capricorn Scientific), and 100 units/mL of streptomycin/penicillin (Sigma-Aldrich), 1% sodium bicarbonate (NaHCO_3_, Gibco) and 2mM L-glutamine (Sigma-Aldrich). rUSUV-Af3 virus was derived from a full-length recombinant clone of the above USUV Af-3-NL isolate, as previously described (Genbank Accession PQ041659.1) [24]. The YF-17D virus stock was derived from a YF-17D full length recombinant clone pACNR-Sp6-YF-17D [25]. Infectious virus titres were determined by plaque assay on BHK-21 J cells.

Cell lines were maintained at 37 °C in a 5% CO_2_ incubator. Vero CCL-81 cells (identity confirmed via in-house STR; LUMC reference VeroMM-2) and A549 cells (identity confirmed via STR) were cultured in DMEM supplemented with 8% FCS and 100 units/mL of streptomycin/penicillin. BHK21-J cells [26] were cultured in Glasgow’s MEM (GMEM, Gibco) supplemented with 8% FCS, 10% tryptose phosphate broth (Gibco), 10mM HEPES (Lonza), and 100 units/mL of streptomycin/penicillin. Human brain microvascular endothelial cells (BMECs, Cell systems, obtained from Erasmus Medical Center) were maintained in MV2 medium (Promocell) prepared according to the manufacturer’s instructions and were used until passage 12.

### Recombinant USUV cDNA clones

Mutant rUSUV-FVR virus was generated using a transformation-associated recombination (TAR) cloning protocol in yeast using a previously described method [24], adapted from Thi Nhu Thao et al., 2020. Briefly, the rUSUV-WT recombinant clone was used as a template to obtain six overlapping PCR fragments covering the length of the USUV genome in the pCC1BAC-his3 vector (Primers in supplemental Table 1a). The PCR fragment containing the envelope region of the genome was cloned into the pCR™8/GW/TOPO vector (Thermofisher) and the nucleotide changes required for the three amino acid mutations were inserted by site-directed mutagenesis (Primers in supplemental table 1b). The purified PCR fragments were then assembled in *S. cerevisiae* according to the TAR recombineering protocol previously described. Correctly assembled recombinant DNA clones, confirmed by Sanger sequencing, were transcribed into RNA and launched using BHK21-J cells. Working virus stocks were grown on Vero CCL-81 cells, and RNA isolated from the passage 4 virus stocks was sequenced by NGS to confirm the presence of the mutations and absence of other (undesired) mutations.

**Fig. 1:**
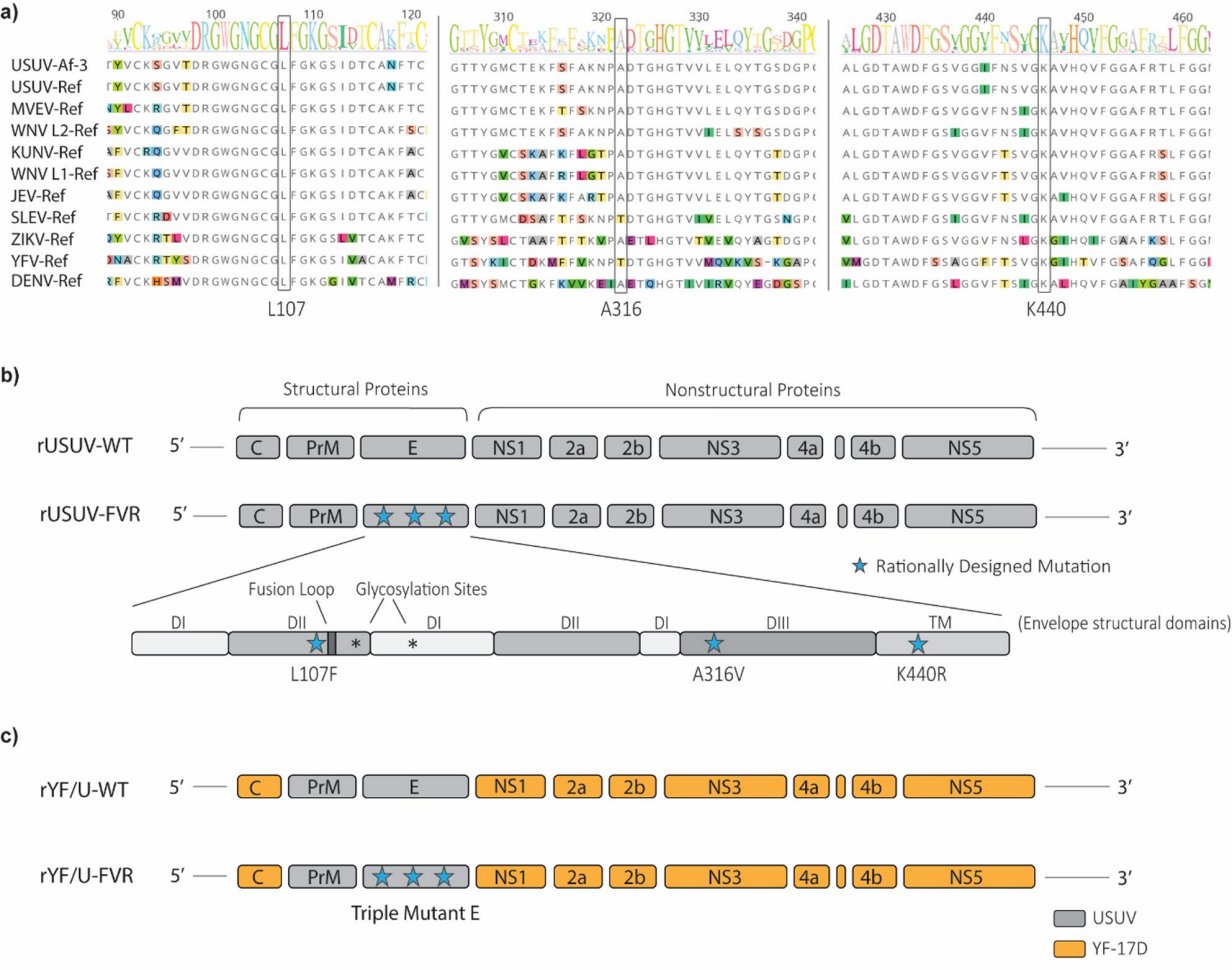
Construction of USUV envelope mutant and YF-17D/USUV chimeric viruses. **a**) Amino acid sequence alignments of USUV-Af3 (NL 2016 strain) and selected related orthoflaviviruses with the sites of introduced amino acid substitutions in E indicated. The consensus sequence is depicted in sequence logo style above the alignment. In each sequence the disagreements to the consensus are highlighted. Genbank reference numbers for sequences used in the alignment can be found in Supplemental Table 4. **b**) Schematic of the USUV genome, depicting the encoded polyprotein with the locations of the structural proteins (C = capsid, PrM = pre-membrane, E = envelope) and non-structural (NS) proteins (NS1, NS2A, NS2B, NS3, NS4A, NS4B and NS5). The locations of the three amino acid substitutions for the rUSUV-FVR mutant are depicted with blue stars, and an expanded view of the envelope protein shows the location of the substitutions relative to the structural domains (DI, DII and DIII) and transmembrane (TM) domain of E. (adapted from [29]. **c**) Schematic of the YF-17D (yellow)/USUV-PrME (grey) chimeric vaccine candidates incorporating either the wild type (WT) or mutant USUV envelope proteins.

rYF/U-WT, and rYF/U-FVR chimeric viruses were also assembled in the pCC1BAC-his3 vector using TAR recombineering. The overlapping PCR fragments were re-designed to overlap the USUV PrM and E genes with the YF-17D capsid and non-structural genes, thus replacing the YFV PrM and E (Supplemental Fig. 1). Primers used for this design strategy are listed in supplemental table 1c. A YF-17D full length recombinant clone pACNR-Sp6-YF-17D [25] was used as a template for obtaining the YFV fragments. The alignments and consensus depicting the clone design in Fig. 1 were made in Geneious version 10.2 (Biomatters).

### Viral growth kinetics

Cells were grown to 80% confluency in multi-well plates. Medium was removed and Vero CCL-81 or BHK21-J cells were infected with virus stock at MOI 0.01, and BMEC cells at MOI 1 for 1 h at 37 °C. After removal of the inoculum, cells were gently washed three times with PBS. The cells were incubated at 37 °C in DMEM supplemented with 8% FCS and 100 units/mL of streptomycin/penicillin, 1% NaHCO3, and 2mM L-glutamine (Vero CCL-81 and BHK21-J cells) or MV2 media (BMEC cells), and supernatant was collected at the specified timepoints to measure the virus titre. We observed large differences in titres of the YF-17D virus and the USUV viruses when titres were determined by plaque assay (PFU/ml) compared to by TCID50 assay. Thus, for the sake of comparability, virus dilutions for infections were based on TCID50 titres and growth curves were also analysed by TCID50 assay.

### Virus quantification

Viral RNA copies were quantified as previously described [24]. In brief, RNA was isolated using the Bio-on-Magnetic-Beads (BOMB) method [28] and RNA copy numbers were determined by reverse transcriptase quantitative PCR (RT-qPCR) with primers targeting either USUV or YFV, and the respective reference standard. A CT cut-off of 35 was set for analysis and this was used to determine the limit of detection. The RT-qPCR primers used are listed in Supplemental Table 1d. Plaque assays were performed on BHK21-J cells, and plaques were counted 4 days after inoculation (BHK21-J cells were used as USUV did not cause clear/countable plaques on Vero CCL-81 cells). The detection limit was 20 pfu/ml for 6-well clusters, or 40 pfu/ml when 12-well clusters were used. Plaque size was measured as the diameter of an approximated circle of the plaque, and an average of this was taken for all plaques in a single well. TCID50 titrations were performed on Vero CCL-81 cells, with CPE assessed at day 5. The detection limit of this assay (with a 1:10 initial dilution) was 31.6 TCID50/ml. Specific infectivity (the number of RNA copies per infectious unit) was determined by dividing the number of viral RNA copies per ml (determined by RT-qPCR) by either the number of pfu/ml (determined by plaque assay) or TCID50/ml. These determinations were performed on the same sample and were performed in triplicate.

### Mouse studies

Ifnar^−/−^ mice on a C57BL/6 background (B6(Cg)-Ifnar1 < tm1.2Ees>/J) were bred and maintained in regulated pathogen free facilities at the LUMC Central Animal Facility (PDC) as previously described [24].

Mice were arranged into groups as follows: for the rUSUV-FVR experiment − 8 weeks old male and female mice in groups of *n* = 8 for virus infections or *n* = 3 for mock controls (experiment 1), for the chimera dose assessment studies – 4 week old female mice (experiment 2) or 6–7 week old male mice (experiment 3) in groups of *n* = 3. Mice were acclimated to the experimental facility for 7 days before inoculation with 100 µl of the specified dose (calculated by pfu/ml) of each respective virus in DMEM, or DMEM alone via sub-cutaneous (SC) injection into the hind flank. Plaque assays and/or TCID50 assays were performed with the virus inoculum to confirm that the titre corresponded to the intended dose for each animal experiment (Supplemental Table 2).

Mice were weighed and monitored daily for the following clinical symptoms: activity, coat condition, gait, hind limb function, and ocular discharge. Sera from tail vein bleeds were collected on alternating days. Mice were euthanized by CO_2_ upon reaching defined humane endpoints, or at the end of the experiment, at day 14. A final serum sample was taken by heart puncture and brain tissue was weighed before being placed in viral transport medium (VTM, MEM without L-glut & HEPES Buffered, 100 units/mL of streptomycin/penicillin, 1x Amphotericin B, 1x Gentamycin, 10% Glycerol) and stored at −80 °C. For virus quantification, tissues were processed as previously described [24]. RNA was isolated and viral load was determined by RT-qPCR, and infectious titres of RT-qPCR positive samples were determined by plaque assay or TCID50 assay as described in 3.4 virus quantification. Infectious virus in serum samples from mouse bleeds was inactivated with 0.2% triton-x, samples were diluted 1:20 and used directly for RT-qPCR.

### Ethics declaration

All experiments involving animals were approved by the Animal Experiments Committee of the LUMC and performed according to the recommendations and guidelines set by the LUMC, the Dutch Experiments on Animals Act, and were in strict accordance with EU regulations (2010/63/EU).

### Statistical analysis

Statistical analyses were performed in GraphPad Prism (version 9). All data are represented as mean ± SEM unless stated otherwise. Survival experiments were analysed using log-rank (Mantel-Cox) test. Replication curves and viral titres in mouse sera were analysed using a one-way ANOVA. Viral titres of mouse tissues were analysed using unpaired t-test corrected for multiple analysis.

## Results

### Construction and stability of an USUV envelope mutant and YF-17D/USUV chimeras

Three amino acids in the E protein of WNV, L107, A316 and K440, that are linked to attenuation, are conserved in orthoflaviviruses, including USUV (Fig. 1a). The L107F, A316V and K440R substitutions (further referred to as FVR) in E were incorporated into USUV using a full length recombinant USUV cDNA clone (rUSUV-WT), site-directed mutagenesis and transformation-associated recombination (TAR) cloning to create rUSUV-FVR (Fig. 1b). NGS of the assembled plasmid confirmed the presence of the three designed mutations (and detected only one additional substitution above a 5% threshold, the silent nucleotide substitution C576T, in the PrM gene (Supplemental table 3a).

Two YF-17D chimeras, with YFV PrM and E replaced by those of wild type USUV (rYF/U-WT) or of the rUSUV-FVR mutant (rYF/U-FVR), were constructed by redesigning the overlapping PCR fragments used for TAR cloning (Fig. 1c, Supplemental Fig. 1). NGS of the correctly assembled plasmids was performed in order to select constructs in which no additional mutations were present (Supplemental table 3b and 3c).

To assess the stability of each of the clone-derived viruses, they were serially passaged four times and passage four (P4) stocks were subjected to NGS (Supplemental Table 3). While some low frequency (5–15%) insertions and point mutations were detected, no amino acid substitutions were detected at a frequency higher than 10% and the three envelope mutations were present in > 99% of reads for both the rUSUV-FVR and rYF/U-FVR viruses.

### The rUSUV-FVR mutant is not significantly attenuated in cell culture

**Fig. 2:**
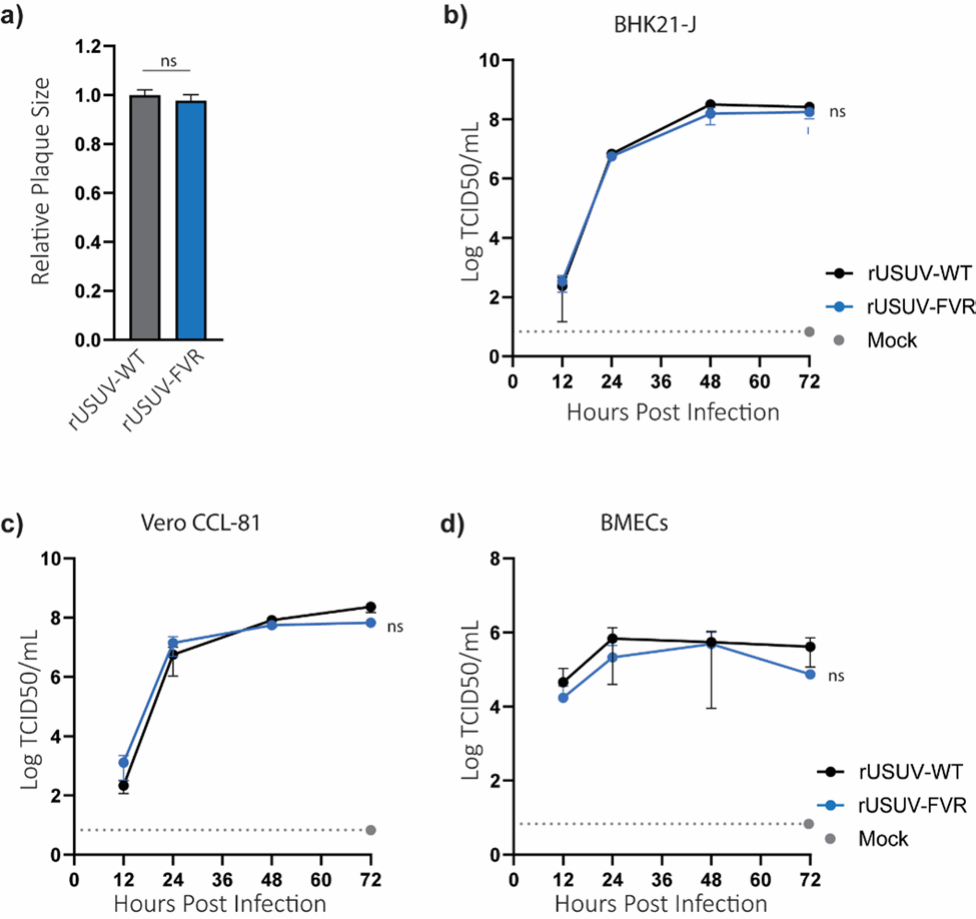
Plaque morphology and growth kinetics of the rUSUV-FVR mutant compared to parental rUSUV. **a**) Plaque diameter was measured, and relative plaque size was calculated, normalized to the average plaque diameter of rUSUV-WT (1). Statistical analysis was performed using unpaired t-test. Replication kinetics of rUSUV-WT and rUSUV-FVR in **b**) Vero CCL-81, **c**) BHK21-J cells and **d**) Human brain microvascular endothelial cells (BMECs). Titres were determined by TCID50 on cell culture supernatants harvested at the specified time points. Statistical analysis was performed using one-way ANOVA

While the rUSUV-FVR virus grew to titres slightly lower than observed for rUSUV-WT (5 × 10^5^ compared to 3 × 10^6^ pfu/ml) no difference in plaque phenotype was observed (Fig. 2a). The replication of the USUV-FVR mutant in BHK21-J cells and Vero CCL-81 cells was similar to that of the wild type virus (Fig. 2b and c). To compare these viruses in a cell line model more relevant with respect to their neuroinvasive properties, we assessed their replication in a primary human brain endothelial cell line (Fig. 2d). Titres of the mutant virus were slightly reduced in this cell line compared to the wild type, but this was not statistically significant. The lack of differences in phenotype of the mutant compared to wild type in these in vitro assays was not surprising considering earlier studies of similar sets of mutations in WNV and JEV [6, 30, 31], although they did not assess this specific combination of mutations (FVR) [19, 20]. Therefore, we continued to compare FVR mutant and WT USUV in a mouse model, hypothesizing that the envelope mutations might impact viral fitness only in the context of an in vivo infection model.

### rUSUV-FVR decreased average mouse survival time compared to rUSUV-WT

**Fig. 3:**
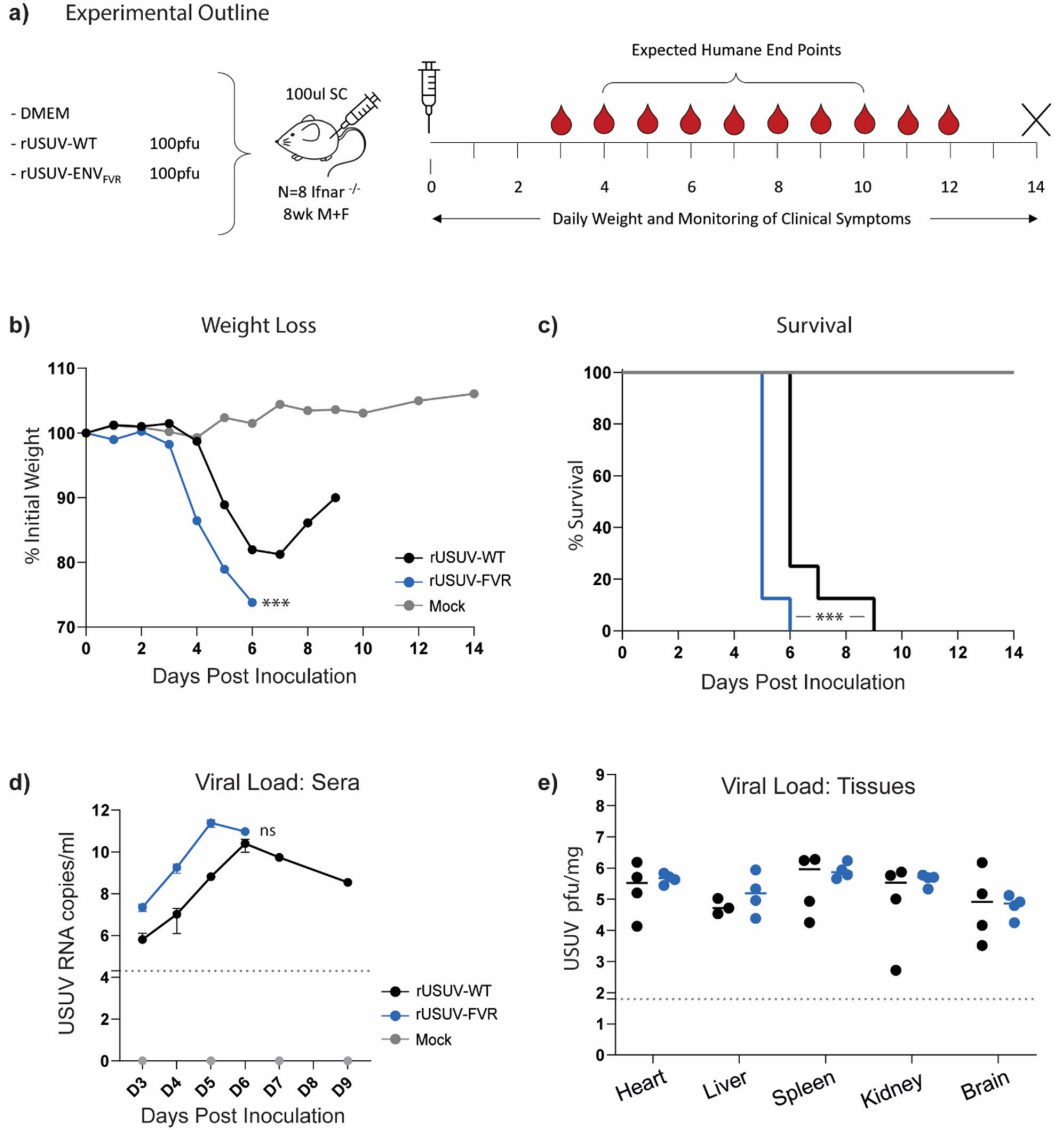
Characterization of rUSUV-FVR in an Ifnar^-/-^ mouse model. **a**) Ifnar^−/−^ mice were inoculated SC with 100pfu/mouse of clone-derived rUSUV-WT virus, rUSUV-FVR mutant, or DMEM alone. Animals were weighed daily and half the mice per group were tail bled on alternate days. Animals were euthanised when they reached humane endpoint, final bleeds were taken by heart puncture, and relevant tissues were harvested. **b**) Average daily weight loss, displayed as percentage of initial weight, for each of the experimental groups. Statistical analysis was performed using a mixed-effects models with Geisser-Greenhouse correction. **c**) Kaplan–Meier curve showing percentage survival for each of the experimental groups. Statistical analysis was performed using the log-rank (Mantel-Cox) test. **d**) Mean (+/-SD) viral titre of tail bleeds and final heart bleed sera from control and mutant virus infected mice, measured by RT-qPCR and absolute quantification using a reference standard. **e**) USUV titre in pfu/mg of homogenised tissues harvested at humane endpoint (HEP), for four mice per group. Statistical analysis was performed using the Mann-Whitney test. Limit of detection represented as dotted grey line. * *P* < 0.05, ** *P* < 0.01, *** *P* < 0.001, **** *P* < 0.0001

Ifnar^−/−^ mice were inoculated subcutaneously with 100 pfu/mouse of rUSUV-FVR or rUSUV-WT, or DMEM as control (Fig. 3a). Unexpectedly, infection with the rUSUV-FVR virus resulted in an earlier onset of clinical symptoms – with mice showing weight loss by day four and reduced activity levels by day five, approximately one day earlier than rUSUV-WT-infected animals (Fig. 3b; Table 1). Consistent with this, most of the rUSUV-FVR-inoculated mice reached humane endpoint (HEP) by day five, while rUSUV-WT infected mice survived until day six or longer (Fig. 3c), as has been observed previously [32]. Viral RNA copies in serum samples from rUSUV-FVR infected mice displayed peak titres approximately one log higher, and one day earlier compared to control mice, though this was not statistically significant (Fig. 3d). Infectious virus titres measured in the homogenised tissue samples were similar between the groups (Fig. 3e).

**Table 1:** Median day of symptom onset for rUSUV- FVR and rUSUV-WT infected Ifnar^-/-^ mice

Virus	Clinical Symptom			
	Lethargic^1^	Hunched Posture	Limping^2^	Ocular Discharge^3^
rUSUV-WT	Day 6	Day 6	Day 5.5	Day 6
rUSUV-FVR	Day 5	Day 5	Day 5	Day 5

^1^ Scored when animals were no longer running around cage unprompted. ^2^ Limp developed in the inoculated hind limb. ^3^ White discharge in one or both eyes resulting in partial or full closure of eye

Back-titration of the virus inoculum showed that rUSUV-FVR-infected animals received a slightly higher dose than rUSUV-WT-infected animals (61pfu/mouse and 38pfu/mouse respectively). However, based on our previous dosing studies inoculating Ifnar^−/−^ mice with doses of 100pfu or higher of rUSUV-WT, this small difference in titre is not enough to explain the difference in the average survival time observed – lethality before day 6 was previously only observed at doses of 1000 pfu/mouse or higher [24]. Sanger sequencing of RT-PCR products generated to amplify USUV sequences from pooled heart homogenate samples confirmed the presence of the envelope mutations in the rUSUV-FVR infected mice.

Another explanation for the unexpected apparently higher virulence of the FVR mutant could be that the effective (mouse) infectious dose of the FVR mutant was underestimated. Mice were inoculated with virus doses that were based on infectious titres that were determined in vitro on cell lines, which may not accurately reflect the infectivity in the animal. We therefore also calculated the number of viral RNA copies that were dosed into mice for each virus, and we determined the specific infectivity of the two viruses (Table 2). rUSUV-FVR infectious titres were slightly lower than that of rUSUV-WT on both BHK and Vero CCL-81 cells, however the number of viral RNA copies per ml were slightly higher. Therefore, the specific infectivity of the rUSUV-FVR mutant is approximately 10-fold lower compared to WT (i.e. the same number of RNA copies yields ~ 10 fold less infectious FVR virus than WT virus, in cell culture). The mice therefore received approximately 10-fold more RNA copies of the FVR virus, i.e. 3.1 × 10^7^ copies, compared to the 3.6 × 10^6^ copies for rUSUV-WT. The specific infectivity within the in vivo model however is not known, but may be different than that measured in vitro.

**Table 2:** RNA copy numbers and infectious virus titres measured by two methods (titrated on BHK21-J and Vero CCL-81 cells) for the recombinant Usutu viruses used in this study

Virus:	Pfu/ml (BHK21-J)	TCID50/ml (Vero CCL-81)	RNA copies/ml (RT-qPCR)	RNA copies/Pfu	RNA copies/TCID50
**rUSUV-WT**	3.2 × 10^6^	3.8 × 10^8^	1.1 × 10^11^	3.6 × 10^4^	3.0 × 10^2^
**rUSUV-FVR**	5.4 × 10^5^	5.3 × 10^7^	1.7 × 10^11^	3.1 × 10^5^	3.2 × 10^3^

### The rYF/U-FVR chimera is more attenuated in vitro than rYF/U-WT

**Fig. 4:**
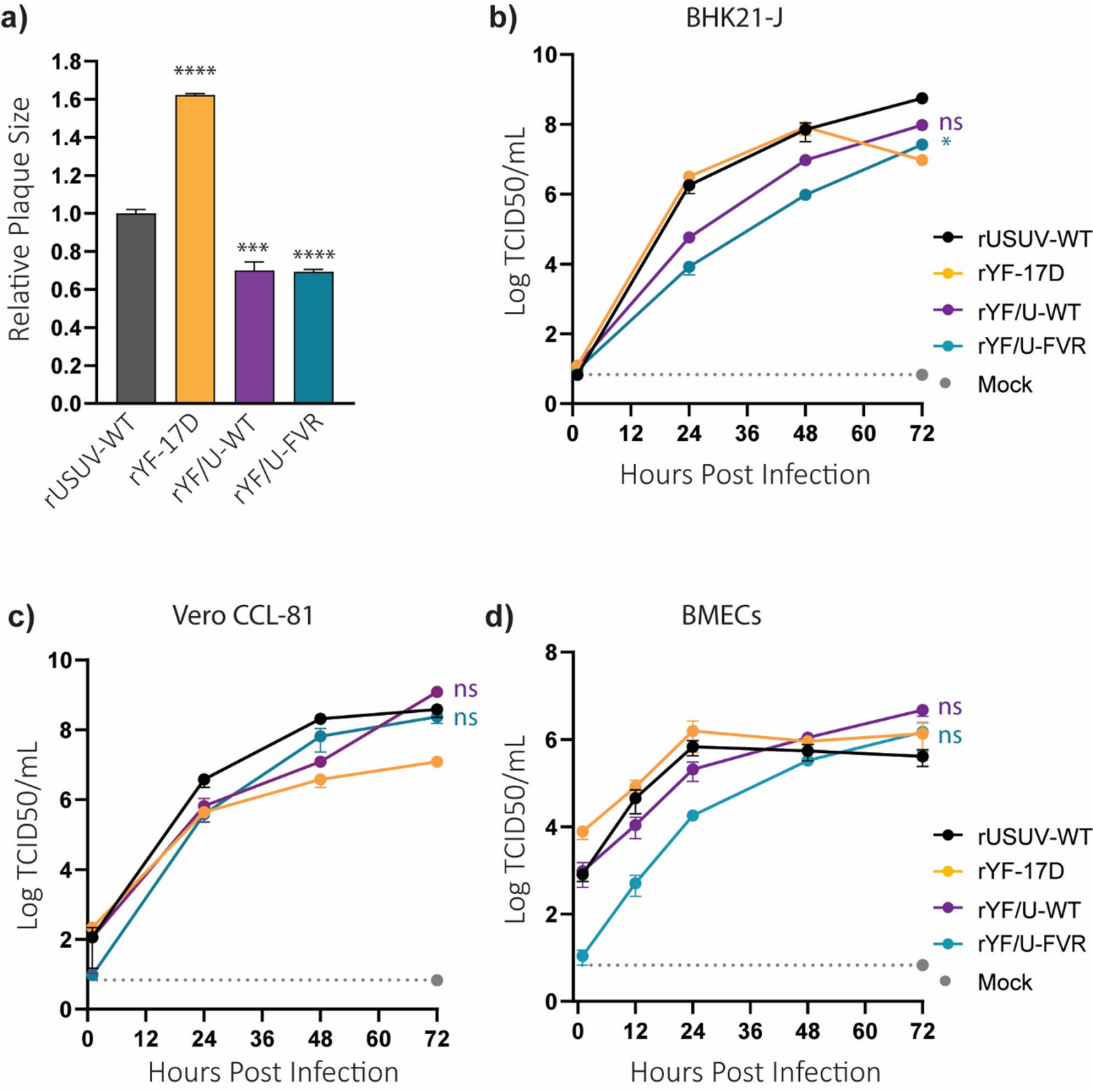
Plaque phenotype and growth kinetics of rUSUV, rYF-17D, and rYF/U chimeric viruses with either the WT or FVR mutant USUV PrME region. In vitro characterisation of rYF/U-WT and rYF/U-FVR compared to rUSUV-WT and rYF-17D. **a**) Plaque diameter was measured, and relative plaque size was calculated against the average of the rUSUV-WT virus plaque diameter. Statistical analysis was performed using unpaired t-test. Replication kinetics on **b**) Vero CCL-81 and **c**) BHK21-J cells at MOI 0.01, and **d**) Human brain microvascular endothelial cells (BMECs) at MOI 1. Titres were determined by TCID50 assay on cell culture supernatants harvested at the specified time points. Statistical analysis was performed using one-way ANOVA

Both rYF/U-WT and rYF/U-FVR exhibit a small plaque phenotype on BHK21-J cells compared to YF-17D or rUSUV-WT, but are similar in size to each other (Fig. 4a). While rYF/U-WT grew to similar titres (pfu/ml) as rUSUV-WT virus on these cells, the mutant rYF-FVR virus titre was almost 40 times lower (Table 3). rYF-17D, on the other hand grew to titres ~ 10-fold higher. To facilitate higher-throughput analysis of samples, we titrated viruses for the replication kinetics studies by TCID50 assay on Vero CCL-81 cells. By this measurement method, rYF-17D titres were almost 20 times lower than rUSUV-WT titres, while both the rYF/U-WT and the rYF/U-FVR chimeras had titres more similar to wild type USUV. In order to better understand the differences in apparent infectivity of these viruses caused by the different titration methods, we also measured viral RNA copies/ml and calculated specific infectivity of the viruses for each of the titration methods (Table 3). When comparing the specific infectivity based upon copies per TCID50, the chimeric viruses were both similar to rUSUV-WT. The rYF-17D on the other hand had more than 10-fold higher copies per TCID50 (similar to the expected ~2 × 10^3^ value of commercial vaccine lots 13). When comparing the specific infectivity based upon copies per pfu, the rYF/U-WT had around 5 times higher copies per pfu than rUSUV-WT, while the rYF/U-FVR chimera, which has low infectious titres on BHK cells, has over 50-fold more copies per infectious particle (2.1 × 10^6^ vs. 3.6 × 10^4^) than rUSUV-WT. On the other hand rYF-17D, which has much higher relative titres on the BHK21-J cells, had around 10 fold less copies per pfu than rUSUV-WT. Overall, the specific infectivity of the chimeras appeared to be more similar to USUV than to YF-17D, and these differences are important to take into account in the analysis of the subsequent in vitro and in vivo studies.

Both chimeras showed delayed replication kinetics in BHK21-J cells (MOI of 0.01 TCID50), with the rYF/U-FVR chimera more delayed and reaching lower titres (Fig. 4b). This was in line with the lower titre measured by plaque assay on BHK21-J cells for this virus. In Vero CCL-81 cells there was no clear difference between the two chimeras, and the viruses replicated at rates much closer to that of rUSUV-WT (Fig. 4c). In the primary human endothelial cell line the chimeras had slower initial replication kinetics but reached titres similar to the rUSUV-WT and rYFV virus controls by 48 h, and continued to increase in titre until 72 h, where the controls had plateaued (Fig. 4d). The growth curves were also assessed by titration by plaque assay and RT-qPCR and these results fitted with the titres that were expected based on the differences in specific infectivity of the different viruses (Supplemental Fig. 2). Overall, the rYF/U-FVR mutant chimera showed a slightly less fit phenotype in vitro than the chimera containing the wild type USUV PrME.

**Table 3:** Specific Infectivity of rYF-17D, rUSUV-WT and chimeric viruses on BHK21-J and Vero CCL-81 cells

Virus:	pfu/ml (BHK21-J)	TCID50/ml (Vero CCL-81)	RNA copies/ml (RT-qPCR)	RNA copies/pfu	RNA copies/TCID50
**rYF-17D**	3.2 × 10^7^	1.9 × 10^7^	8.0 × 10^10^	2.5 × 10^3^	4.2 × 10^3^
**rYF/U-WT**	2.2 × 10^6^	5.7 × 10^8^	4.3 × 10^11^	1.9 × 10^5^	7.4 × 10^2^
**rYF/U-FVR**	5.8 × 10^4^	1.3 × 10^8^	1.2 × 10^11^	2.1 × 10^6^	8.9 × 10^2^
**rUSUV-WT**	3.2 × 10^6^	3.8 × 10^8^	1.1 × 10^11^	3.6 × 10^4^	3.0 × 10^2^

### The rYF/U-WT and FVR mutant chimeras both cause lethal infections in an Ifnar^-/-^ mouse model

**Fig. 5:**
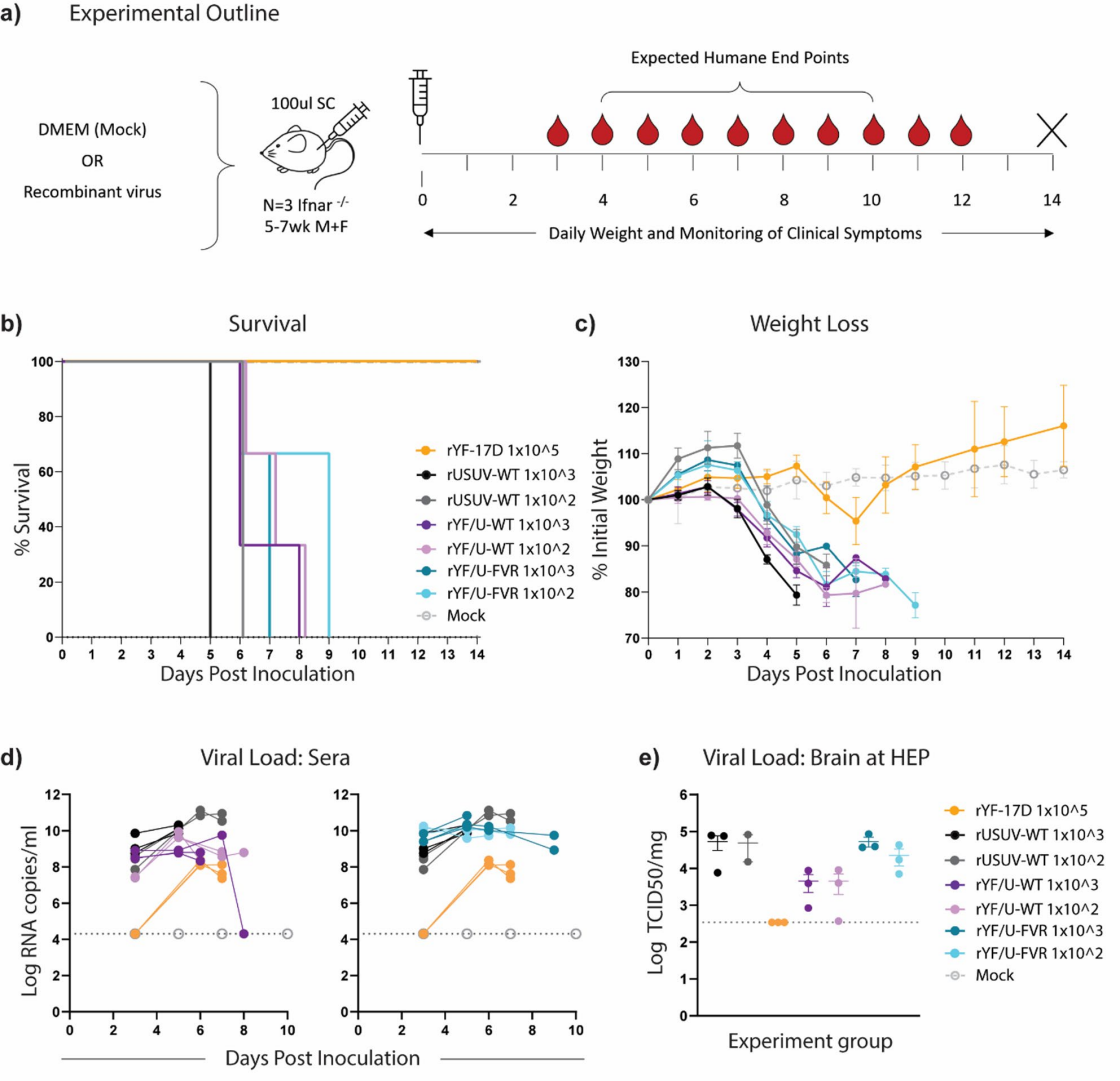
Characterisation of rYF/U-WT and rYF/U-FVR chimeras in an Ifnar^-/-^ mouse model. **a**) Three Ifnar^−/−^ mice per group were inoculated SC with 1 × 10^2^ or 1 × 10^3^ pfu/mouse of rUSUV-WT, rYF/U-WT or rYF/U-FVR, or 1 × 10^5^ pfu/mouse of rYF-17D. Mice were weighed daily and half the mice per group were tail bled on alternate days. Animals were euthanised when they reached the defined humane endpoint, final bleeds were taken by heart puncture and brain tissue was harvested. **b**) Survival rates for each of the experimental groups. **c**) Daily weight loss measured as a percentage of initial weight for each of the experimental groups showing mean ± SD. **d**) USUV RNA copies/ml of tail bleeds and final heart bleed sera from individual mice as measured by RT-qPCR. Chimera-infected mice groups at both doses were each compared to the corresponding control groups. **e**) TCID50/mg of homogenised brain tissues from each group, harvested at humane endpoint or end of experiment (day14). Limit of detection represented as dotted grey lines

The attenuation of the rYF/USUV chimeras with either the WT or FVR mutant USUV PrME region was assessed in a pilot study in Ifnar^−/−^ mice, in comparison to rYF-17D and rUSUV-WT control viruses (Fig. 5a). Based on literature we expected that a chimeric neurotropic virus in this mouse model would still be attenuated even at doses similar to the rYF-17D control [33, 34]. However, at such high doses (1 × 10^5^ and 1 × 10^4^ pfu/mouse) the rYF/U-WT chimera caused lethality by day 6, while the YF-17D mice dosed at 1 × 10^5^ survived with minimal weight loss or clinical symptoms (Supplemental Fig. 3a and 3b, Table 4). Even at much lower doses of 1 × 10^3^ and 1 × 10^2^ pfu/mouse, both the rYF/U-WT and the rYF/U-FVR viruses caused lethal infections in 100% of the animals, with the HEP reached only a few days later than in the rUSUV-WT-infected group (Fig. 5b). Similar to what we observed for rUSUV-WT infection, the lethality correlated closely with onset of weight loss and clinical symptoms, and at the lower doses this disease progression occurred less rapidly (Fig. 5c; Table 4) [24]. At the 1 × 10^3^ and 1 × 10^2^ pfu doses, ataxia or paralysis occurred in one or more of the mice from each of the chimeric virus inoculated groups, indicative of a neuroinvasive infection (Table 4).

The viremia over the course of the infection was measured by RT-qPCR. rUSUV-WT-infected mice showed a similar trend as observed previously (Fig. 3)[24], where early titres were higher in animals that received a higher dose, and peak titre occurred at or just before HEP was reached (Fig. 5d). rYF-17D viremia was more delayed, correlating with the observed weight loss, and reached lower titres in RNA copies/ml. For the chimeric viruses, no clear correlation between the titre and the dose was observed. Surprisingly however, the titres were consistently higher for all animals inoculated with the rYF/U-FVR chimera compared to those that were inoculated the with rYF/U-WT chimera. Infectious titres measured in the brain tissue were also lower in the WT PrME chimera groups than in rYF/U-FVR inoculated animals – which had titres similar to the rUSUV-WT control mice (Fig. 5e).

This lethality was not caused by contamination with WT USUV virus, as serum samples of animals infected with chimeric virus were negative in RT-qPCR using primers targeting USUV NS5 (which do not recognise the YFV sequence that is present in the chimeric viruses), and virus from the harvested brain tissues still exhibited the small plaque phenotype (Supplemental Fig. 4).

**Table 4:** Median day of symptom onset for YF-17D chimera and control infected Ifnar^-/-^ mice

Virus	Dose (pfu/mouse)	Clinical Symptom				
		Lethargic^1^	Hunched	Hind limb limp^2^	Ocular Discharge^3^	Ataxia^4a^/Paralysis^4b^
rYF-17D	1 × 10^5^	na	na	Day 7	na	na
rUSUV-WT	1 × 10^3^	Day 5	Day 5	Day 4	Day 5	na
	1 × 10^2^	Day 6	Day 6	Day 4	Day 6	na
rYF/U-WT	1 × 10^3^	Day 5	Day 5	Day 4	Day 6	Day 8 - in 1 of 3 mice^4a^
	1 × 10^2^	Day 5	Day 5	na	Day 7	Day 8 - in 1 of 3 mice^4b^
rYF/U-FVR	1 × 10^3^	Day 5	Day 6	na	Day 6	Day 7 - in 1 of 3 mice^4a^
	1 × 10^2^	Day 6	Day 6	na	Day 6	Day 9 - in 2 of 3 mice^4b^

^1^ Score when animals were no longer running around cage unprompted. ^2^ Limp or disfavouring of the inoculated hind limb. ^3^ White discharge in one or both eyes resulting in partial or full closure of eye. ^4a^ Mouse was wobbly and uncoordinated, or 4^b^ developed para or tetraplegia

## Discussion and conclusion

Vaccine design strategies based on established vaccine platforms may enable faster development of safer live attenuated vaccine candidates against (re)emerging orthoflaviviruses. Here, we used a chimeric virus design with the YF-17D LAV backbone as a potential vaccine candidate against USUV. Based on previous studies using chimeras in this platform, we anticipated that additional attenuating mutations in the USUV E protein might be required to ensure vaccine candidate safety. A set of three mutations (denoted as FVR) in the E protein, previously associated with attenuation in JEV and WNV vaccines, were therefore assessed both in USUV itself, and in a YF-17D/USUV chimeric vaccine candidate. Surprisingly, insertion of these mutations into the USUV genome seemed to increase the lethality of the virus in an Ifnar^−/−^ mouse model, and did not markedly impact survival when included in the YF-17D/USUV chimera.

The increased lethality of the rUSUV-FVR mutant was unexpected. Although context-specific effects of such mutations have been described, they usually result in attenuation or unchanged virulence rather than increased pathogenicity. For example, incorporating the SA14-14-2 JEV envelope mutations into a highly virulent JEV strain did not attenuate the virus [35], and the individual L107F, A316V and K440R mutations reduced neurovirulence of the YF-17D/WNV-PrME chimera, but not of the NY99 WNV strain [19, 20]. Similarly, the E138K mutation attenuated a YF-17D/JEV-PrME chimera but not a YF-17D/WNV-PrME chimera or virulent WNV strain, although it did attenuate a low-pathogenic WNV variant while showing reversion [14, 20, 31, 36, 37]. None of these earlier studies however reported increased pathogenicity. We consider four possible explanations for our observations: (1) differences between specific infectivity in vitro and in vivo; (2) the higher number of viral RNA copies in the mutant virus inoculum; (3) structural differences in the USUV E protein compared to other orthoflaviviruses could contribute; (4) use of an immunocompromised animal model. These potential explanations are further discussed below.

The rUSUV-FVR mutant had more viral genome copies per infectious unit (pfu or TCID50) than rUSUV-WT. Consequently, mice inoculated with equal pfu doses measured in plaque assays in cell culture received ~ 10-fold more viral RNA copies (‘particles’;, i.e. 3.1 × 10^7^ vs. 3.1 × 10^6^) of rUSUV-FVR compared to rUSUV WT. If in vitro determined infectivity (pfu) of particles (genome copies) does not represent the in vivo situation, rUSUV-FVR–inoculated mice may effectively have received a higher infectious dose. This would suggest that the FVR mutations do not confer a phenotype in vivo, and that the more rapid lethality could be attributed to the higher dose of infectious particles that was used in the USUV-FVR infected mice. Consistent with this explanation, we have previously shown that mice receiving 1000 pfu/mouse reached HEP between day 5–6 [24], in line with the lethality observed by day 5 for the rUSUV-FVR-infected mice in this study.

Alternatively, the higher viral RNA-to-pfu ratio could also indicate a larger proportion of non-infectious particles in the inoculum used to infect mice. Use of extremely high doses of non-replicating particles caused lethality in a human trial with recombinant adenovirus vector vaccine [13, 38], raising the possibility that increased numbers of non-infectious rUSUV-FVR particles (compared to WT) caused immunopathology and faster death [39]. IFN-independent mechanisms should be involved, as we used IFNAR-/- mice. However, this explanation seems less likely because many studies have demonstrated that non-infectious particles inhibit virus replication, which would be expected to delay rather than accelerate mortality in rUSUV-FVR-infected animals [40, 41].

Sequence conservation among orthoflavivirus E proteins is relatively high (USUV–WNV 77%, USUV–JEV 80%, JEV–WNV 79%), and knowledge of JEV attenuating mutations has previously successfully guided WNV vaccine design. [20, 21]. Therefore, we hypothesized this would be the case for USUV as well. The lack of attenuation in USUV may therefore reflect structural differences in its E protein. For example, the USUV E protein contains an additional glycosylation site at E-118 that is absent in other orthoflaviviruses, which could impact the effect of the FVR mutations. Furthermore, the USUV E protein structure displays asymmetry in the presence of a cysteine bond in the fusion loop (where the L107F mutation is located), and a buried arginine in a conserved motif of the receptor binding domain (location of the A316V mutation) [22, 29]. These structural differences could alter the phenotypic effects of the FVR substitutions. If the rUSUV-FVR mutant is indeed more pathogenic, analysis of the contribution of individual mutations would be interesting.

A final factor that might have contributed to the apparent increased lethality of the rUSUV-FVR mutant, is the immunocompromised mouse model. Previous studies on these mutations in other viruses have used immunocompetent mice. Because USUV does not consistently cause disease in these models, either young mice or immunocompromised mice are required instead [42] - we used Ifnar^−/−^ mice, which lack α and β interferon receptors. These mice are highly susceptible to USUV infection [24, 42] and have been previously used in orthoflavivirus studies [43, 44]. Earlier studies primarily assessed neurovirulence (injecting intracranially), which bypasses the extra-neural impacts of the introduced mutations [20, 30]. Our subcutaneous inoculation model aimed to mimic vaccination and also allowed us to assess neuroinvasion. Previous studies that assessed neuroinvasion used intraperitoneal inoculation [19, 45]. The IP route would be expected to lead to the same or higher levels of lethality than the SC route [46].

Differences in inoculation routes affect the cell types initially infected, and envelope mutations influencing attachment, entry, or fusion might exert stronger effects in certain cell types, potentially altering pathogenicity.

Everything considered, we suspect that the FVR mutations that attenuate WNV do not attenuate USUV in (immune compromised) mice, and that the faster lethality observed with rUSUV-FVR likely reflects the ~ 10 times more particle dose administered.

Another surprising finding of our study was the lethality of the rYF/U-WT and FVR chimeras in the Ifnar^−/−^ mice. Similar vaccines have demonstrated incredibly robust safety profiles and the point of using the YF-17D platform is that this characteristic would be maintained [12]. Firstly, chimerisation itself has been shown to be attenuating [12, 47]. While the YF-17D chimera vaccines for JEV or WNV do contain attenuated PrME regions, incorporating WT JEV or WNV PrME still resulted in significantly attenuated neuroinvasion compared to the parent virus, and similar or reduced neurovirulence compared to the YF-17D virus [15, 20, 21]. Secondly, USUV is a less pathogenic virus than WNV or JEV, with comparatively slower replication kinetics and less neuropathology [48, 49]. Replacing the WNV envelope with that of USUV resulted in a virus that was attenuated compared to WNV, although more lethal than USUV, suggesting that an USUV chimera would also be expected to be more attenuated when containing the WNV envelope [50].

We currently do not have an explanation for the lethality of our YFV-17D/USUV chimeras, but several theories could be further investigated. As discussed above, differences in particle infectivity could influence the effective dose, although this seems unlikely because control mice received ~ 1000-fold more PFU of YF-17D yet a similar number of RNA copies compared with the chimera inocula.

The use of an immunocompromised animal model is also unlikely to (fully) explain the pathogenicity of the chimeras, since Ifnar^−/−^ mice have been shown to survive infections with other chimeras that incorporate PrME from neuroinvasive viruses. For example, these mice survive high doses of a chimera containing WNV PrME in a DENV backbone[34] or a chimera containing the JEV PrME in a Binjari virus platform (an insect-specific orthoflavivirus)[51]. More convincingly, while both the YF-17D and JEV SA14-14-2 LAVS are lethal in AG129 mice (which lack γ interferon receptors in addition to the α and β receptors), the YF-17D/JEV-SA14-14-2-PrME chimera was well tolerated [47].

However, precedent exists for lethal phenotypes of YF-17D chimeras containing neuroinvasive PrME regions. A YF-17D/Modoc virus (MODV) chimera caused neuroinvasion and lethality in SCID mice, despite YF-17D itself being non-lethal (and not neuroinvasive) [7]. Therefore, it is plausible that the combination of the YF-17D backbone with non-attenuated USUV PrME fails to cause sufficient attenuation. To better understand the phenotype of the YFV-17D/USUV chimeras, it is important to characterise them in immunocompetent mouse models, for example using young mice, or by intracranial inoculation of adult mice [42, 52]. Furthermore, direct comparison with established attenuated YF-17D chimeras, like those containing WNV or JEV PrME, would help clarify the contribution of PrME-mediated neuroinvasion to the observed phenotype.

A study assessing another chimeric USUV vaccine candidate, based on the JEV SA14-14-2 backbone, had similar issues in finding an appropriate animal model for safety assessment [53]. This chimeric virus displayed slightly higher neurovirulence than JEV SA14-14-2, and was not neuroinvasive in 4wk old BALB/c mice. However, no direct comparison to USUV was made, so whether the vaccine is attenuated compared to WT virus is unknown. Another, virus-like particle-based USUV vaccine candidate was not lethal in Ifnar^−/−^ mice [54].

Our findings suggest that current (cell-based) in vitro systems may poorly predict in vivo outcomes. More complex and relevant systems like primary skin cells, 3 d models, explants or blood-brain barrier models may have a better predictive value for the in vivo situation[55]. Our study further highlights the importance of developing better, more ethical (animal-free) models, to improve efficiency and translatability of research and minimise the number of animals sacrificed. Furthermore, plaque size does not necessarily correlate with attenuation, further complicating interpretation of in vitro results[56].

The decreased average survival time of rUSUV-FVR-infected mice, the unexpectedly high lethality of the rYF/U-WT and rYF/U-FVR chimeras, and the low numbers of mice used (*n* = 3) for the pilot dose assessment studies make it difficult to conclude whether additional attenuating mutations might actually be required for engineering a safe USUV vaccine. If so, mutations targeting other envelope regions will be important. For example, it could be considered to increase glycosaminoglycan binding, removing the N-glycosylation sites, or mutating other amino acids at the fusion loop or hinge domain of the envelope [11, 32].

In summary, the unexpected lethality of our rUSUV-FVR mutant suggests that the FVR mutations may not attenuate USUV in Ifnar^−/−^ mice, either due to the immunocompromised model or because these substitutions are not attenuating in the USUV context. This highlights the complications and unpredictability of incorporating rationally designed mutations into potential vaccine candidates, in line with several other studies [11, 32, 57]. More biological or advanced AI-based studies on available datasets are required to improve predictions and inform us better on rational design of mutations. Our use of an immunocompromised mouse model could also explain why the incorporation of the PrME region from the normally low pathogenic but neuroinvasive USUV into the YF-17D platform causes lethal infections in these mice. Although the YF-17D/USUV chimeras were lethal in Ifnar^−/−^ mice, this does not preclude their potential as vaccine candidates, only that better/alternative infection models (and possibly additional attenuating mutations in USUV PrME) are required to assess their phenotype and safety.

## Supplementary Information

Supplementary Material 1

## Data Availability

Available upon request.
