# Supplementary material for: A yellow fever 17D and Usutu virus chimera with rationally designed mutations in the envelope protein is lethal in an Ifnar-/- mouse model
Source: Virol J. 2026 Jul 3;23:168. doi: 10.1186/s12985-026-03145-x (PMC13330216; doi:10.1186/s12985-026-03145-x)
Supplement: Supplementary file 1 — Supplementary Material 1 [file 12985_2026_3145_MOESM1_ESM.docx]

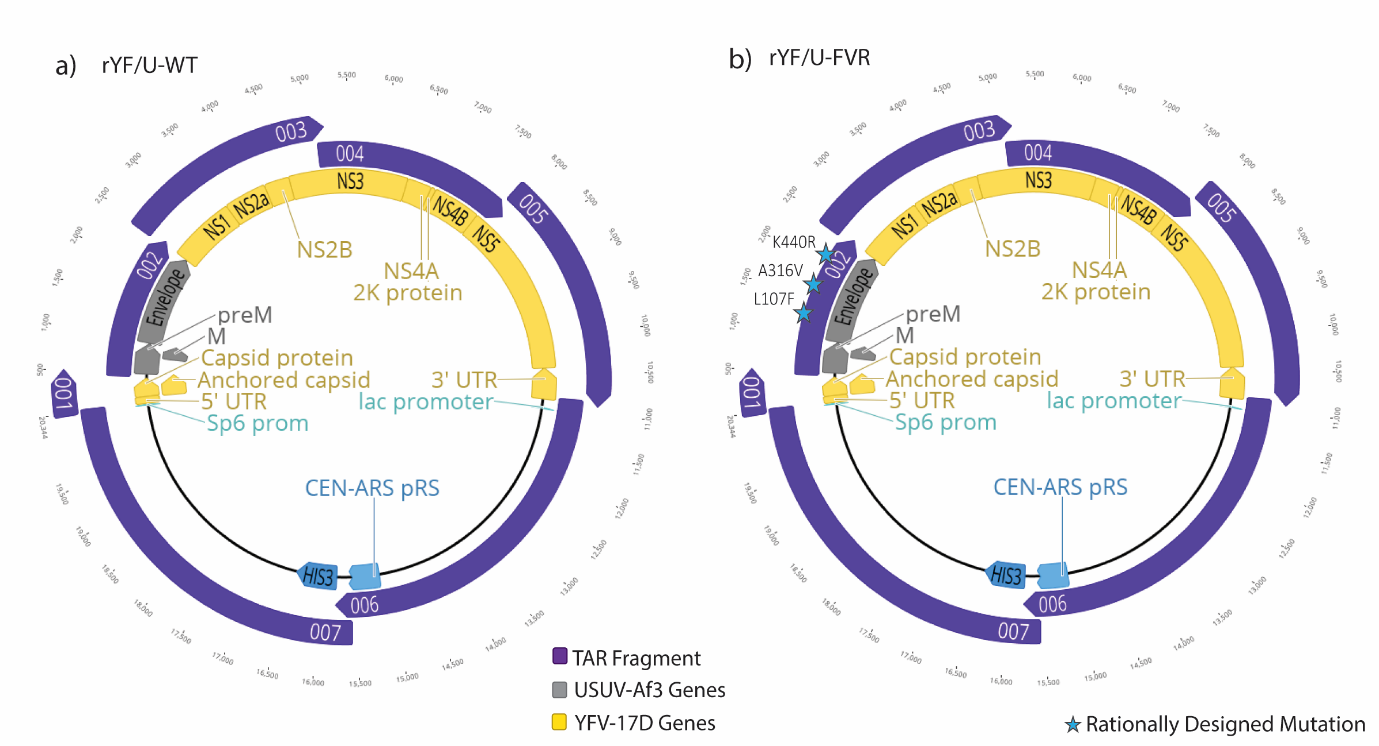


##### Supplemental Figure 1. TAR Recombineering Fragment Design Strategy for YF-17D/USUV Chimeras

The pCC1BAC-his3 plasmid maps showing the TAR design strategy for building YF-17D/USUV chimeras replacing the YF-17D PrME with a) the USUV PrME region or b) the USUV PrME-FVR incorporating 3 mutations in the envelope protein. Overlapping fragments (annotated in purple) were obtained by PCR, using either an rUSUV-Af-3 full length recombinant clone built in an the pCC1BAC-his3 vector, or a rYF-17D full length recombinant clone as the template depending on the section each fragment corresponds to. USUV genes are annotated in grey, YF-17D genes and untranslated regions (UTRs) are annotated in yellow. Features of the pCC1BAC-his3 vector are annotated in shades of blue, and the three mutations incorporated in rYF/U-FVR chimera are represented as blue stars.


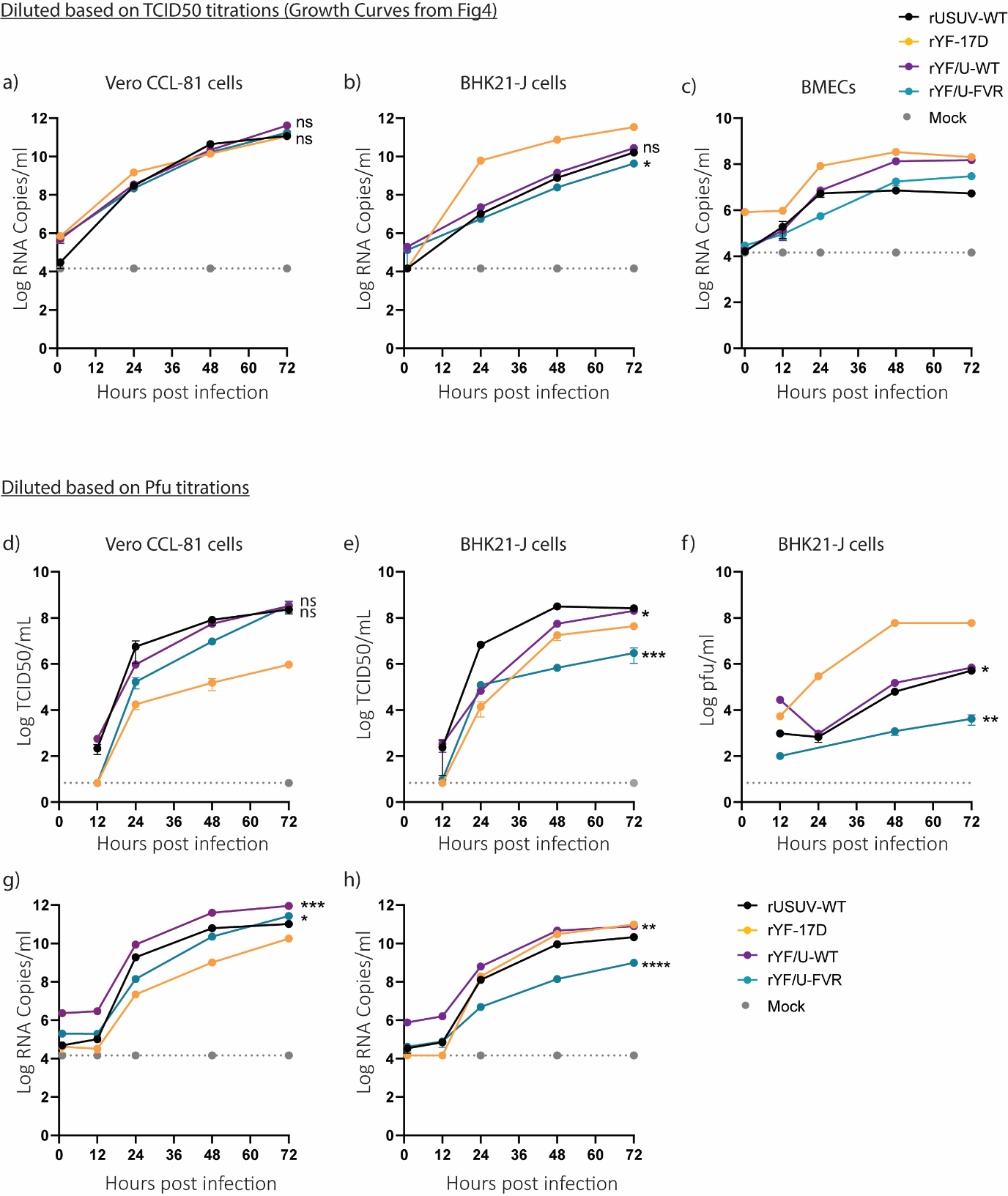


##### Supplemental Figure 2. Growth curves of YFV-17D/USUV chimeric viruses measured by additional titration methods.

Growth curves were assessed by alternative input titrations and alternative measurements of virus titres in the supernatant to provide a more in depth phenotype assessment of the chimeric viruses. Supernatants from growth curves on a) Vero CCL-81 and b) BHK21-J cells infected at an MOI of 0.01 TCID50 or c) BMECs infected at an MOI of 1 TCID50 were measured by RT-qPCR. Measurements by TCID50/ml for these growth curves are shown in Figure4. Supernatants from growth curves on d+g) Vero CCL-81 and e+h) BHK21-J cells infected at MOI 0.01 pfu, were measured by TCID50 assay and RT-qPCR respectively, and f) supernatants from BHK21-J cells were also assessed by plaque assay. Statistical analysis was performed using one-way ANOVA.


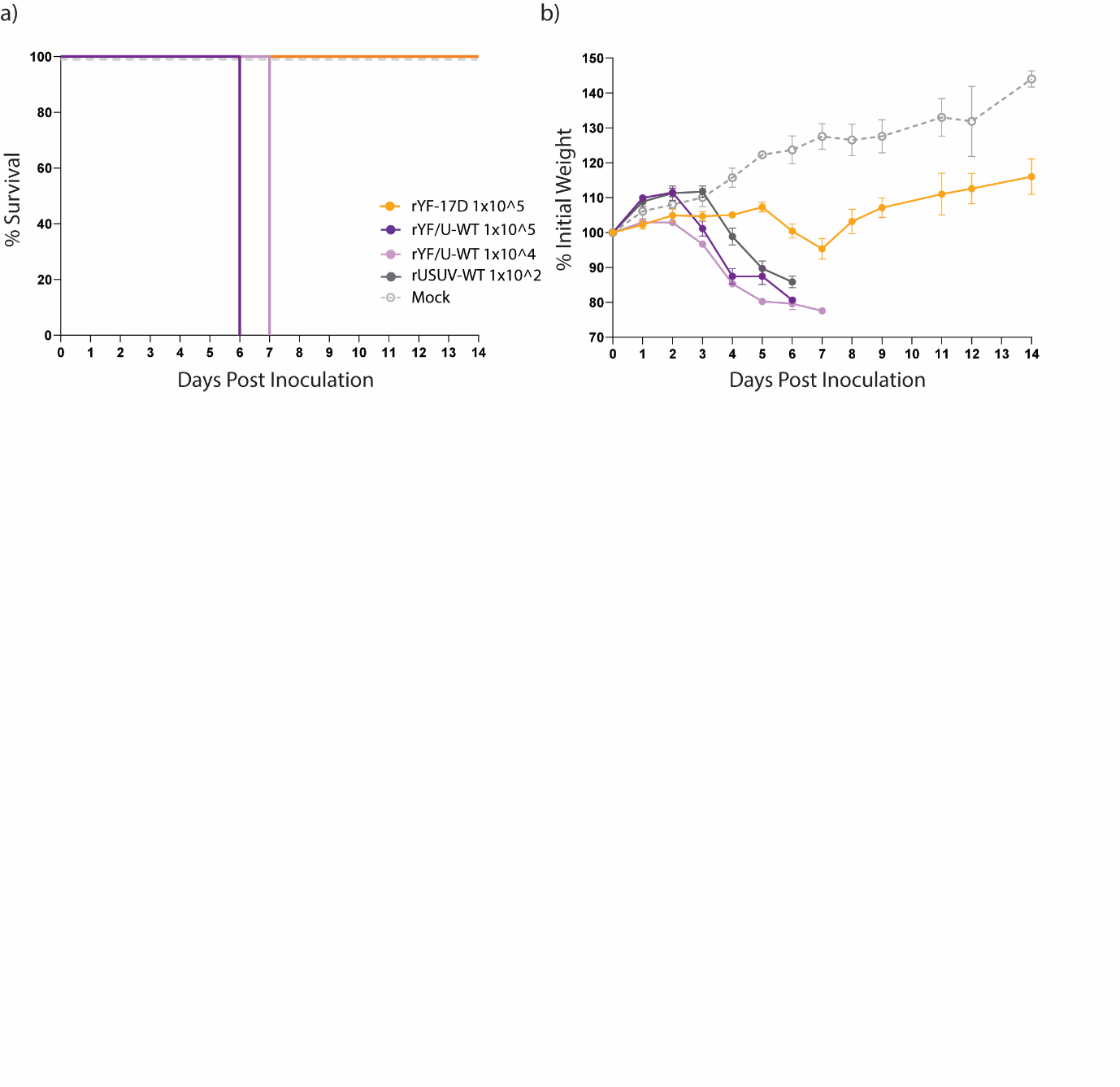


##### Supplemental Figure 3. High dose pilot studies for the YF/U-WT chimera in Ifnar^-/-^ mice.

Ifnar^-/-^ mice were inoculated SC with 1x10^5^ or 1x10^4^ pfu/mouse of rYF/U-WT – control groups shown are as described in figure 5 of the manuscript. Mice were weighed daily and half the mice per group were tail bled on alternate days. Animals were euthanised when they reached humane endpoint, final bleeds were taken by heart puncture and brain tissue was harvested. a) Survival rates for each of the experimental groups. b) Daily weight loss measured as a percentage of initial weight for each of the experimental groups showing mean ± SD. Back titration information in Supplemental Table 2.


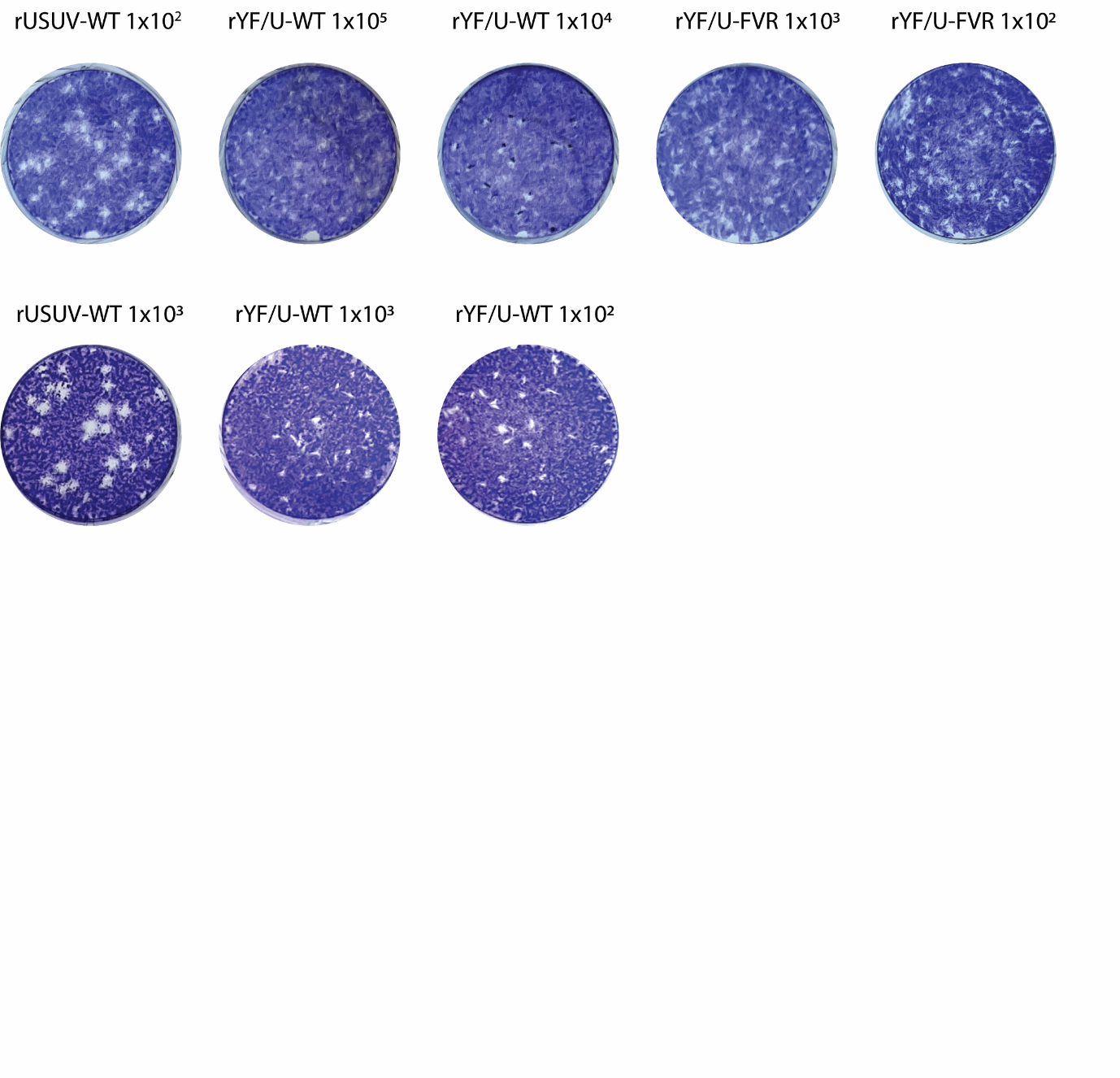


##### Supplemental Figure 4. Plaque phenotypes of viruses from homogenised brain tissue

Plaque assays on BHK21-J cells were performed for homogenised brain tissues harvested at HEP from Ifnar^-/-^ mice inoculated with respective doses of rUSUV-WT, rYF/U-WT or rYF/U-FVR. Images are of selected wells representative of the plaque assay size for each group.

##### Supplemental Table 1. Primers used in this study

a) Primers used to PCR rUSUV-FVR TAR Recombineering Fragments

|  | Forward (5’-3’) | Reverse (5’-3’) |
| --- | --- | --- |
| Fragment 1 | **ACGCCAGGGTTTTCCCAGTCACGAC**GCGGCCGC*ATTTAGGTGACACTATAG*AGATGTTGGCCTGTGTGAG | CCAGATCACTTTCAACAACG |
| Fragment 2 | CATGCACGTGGCCTGAAAC | GTCAATGACTCTGCTGGCC |
| Fragment 3 | CCGCAAGTCCTATGACACG | CAAACGGAGTGGTGTCAG |
| Fragment 4 | GCAATTCTCAACGTGACTACC | **GCCTGCAGGTCGACTCTAGAGGATC**CTTAAGAGATCCTGTGTTCTTCTCCA |
| Fragment 5 | GGCTGATGGGGAACAAGACCACAGGATCTCTTAAG**GATCCTCTAGAGTCGACCTGC** | **GCCTTCGTTTATCTTGCCTGCTC** |
| Fragment 6 | **CCATCATTAAAAGATACGAGGCGCGTGT** | GCTCACGCAGACAAACGACT*CTATAGTGTCACCTAAAT*GCGGCCGC**GTCGTGACTGGG** |

^Key: In bold vector sequence, in italics the SP6 promoter sequence, underlined USUV sequence.^

b) Primers used in rUSUV-FVR mutant site directed mutagenesis

| **Gene** | **Amino Acid Change(s) – Gene #** | **Nucleotide Change(s) – Genome #** | **Sequences (Fwd/Rev)** |
| --- | --- | --- | --- |
| E | L107F | C1294T, A1296T | GGGGCAATGGCTGTGGATTTTTTGGCAAAGGAAGTATAG  CTATACTTCCTTTGCCAAAAAATCCACAGCCATTGCCCC |
|  | A316V | C944T, T945C | CTTTTGCAAAAAATCCGGTCGACACGGGTCACGGCAC  GTGCCGTGACCCGTGTCGACCGGATTTTTTGCAAAAG |
|  | K440R | A1315C, A1316G | GATTTTCAACTCTGTAGGGCGGGCGGTGCATCAGGTC  GACCTGATGCACCGCCCGCCCTACAGAGTTGAAAATC |

c) Primers used to PCR YF/U-WT and YF/U-FVR chimera TAR Recombineering Fragments

|  | Forward (5’-3’) | Reverse (5’-3’) |
| --- | --- | --- |
| Fragment 1 | **AGTCACGAC**GCGGCCGCA*TTTAGGTGACACTATAG*AGTAAATCCTGTGTGCTAATTGAGGTGCATTGGTC | TCATGACTTTCCCCTGGAAGTTGGAAAGCTTTAATCCACCCGTCATCAACAGCATTCCCAAAATTAGGAA |
| Fragment 2 | TTCCTAATTTTGGGAATGCTGTTGATGACGGGTGGATTAAAGCTTTCCAACTTCCAGGGGAAAGTCATGA | TCTCTCTTGCCAAAGTTGATGGCGCATCCTTGATCTGCATGGACGTTTGTGGCGAGAAAGAGAAGCACCC |
| Fragment 3 | GGGTGCTTCTCTTTCTCGCCACAAACGTCCATGCAGATCAAGGATGCGCCATCAACTTTGGCAAGAGAGA | GCTCCAGGATGAAAATCAAGGAC |
| Fragment 4 | CCGCCATATCCCAGACTGAGGTG | TTTGACCCCACTCACTTCC |
| Fragment 5 | CGCAAAGTTAAGGTGGTTCC | **CTTGCATGCCTGCAGGTCGACTCTAGAGGATC**CTTAAGTGGTTTTGTGTTTGTCATCCAAAGGTCTGCTT |
| Fragment 6 | AAGCAGACCTTTGGATGACAAACACAAAACCACTTAAG**GATCCTCTAGAGTCGACCTGCAGGCATGCAAG** | **GCCTTCGTTTATCTTGCCTGCTC** |
| Fragment 7 | **CCATCATTAAAAGATACGAGGCGCGTGT** | GACCAATGCACCTCAATTAGCACACAGGATTTACT*CTATAGTGTCACCTAAAT*GCGGCCGC**GTCGTGACT** |

^Key: In bold vector sequence, in italics the SP6 promoter sequence, underlined YFV sequence, grey highlight USUV sequence.^

d) Primers used in qRT-PCR protocols

| **Primer** | **Sequence** |
| --- | --- |
| USUV _Fwd | TCAGAAAAGACGTGCCAGAG |
| USUV_Rev | AAAGTCCTTCCGTCCTTCATG |
| USUV_Probe_FAM | CCTGAAAGTGGTTTGAGCAGAAAGGC |
| YFV_Fwd | GCACGGATGTGACAGACTGAAGA |
| YFV_Rev | CCAGGCCGAACCTGTCAT |
| YFV_Probe_FAM | CGACTGTGTGGTCCGGCCCATC |
| EAV_Fwd | CATCTCTTGCTTTGCTCCTTAG |
| EAV_Rev | GCTTTGCCATTGGGTTGATACC |
| EAV_Probe_TQ-CY5 | CGCTGTCAGAACAACATTATTGCCCAC |

##### Supplemental Table 2. Titration of Viral inoculum used in animal experiments

a) Back titrations for rUSUV-FVR experiment *(dosing performed based on pfu/ml titre of the respective virus stocks and back titrated by plaque assay. Equivalent TCID50 titre stated for comparison to chimera experiments).*

| **Experiment One:**  **Group** | **Expected PFU/mouse*** | **Measured PFU/Mouse** |
| --- | --- | --- |
| rUSUV-WT | 1E+03 | 38 |
| rUSUV-FVR | 1E+03 | 61 |

*equivalent to 1.4x10^3 TCID50/ml

b) Back titrations for YF/U-WT chimera experiments (dosing performed based on pfu/ml titre of the respective virus stocks and back titrated by plaque assay)

| **Experiment Two:**  **Group** | **Expected titre/mouse** | | **Measured titre/mouse** | |
| --- | --- | --- | --- | --- |
|  | **PFU** | **TCID50** | **PFU** | **TCID50*** |
| rUSUV-WT 1x10^2^ | 1x10^2^ | 1.2x10^4^ | 1.4 x10^2^ | 3.2x10^1^ |
| rYF-17D 1x10^5^ | 1x10^5^ | 5.9x10^4^ | 1.5 x10^5^ | 3.2x10^3^ |
| rYF/U-WT 1x10^5^ | 1x10^5^ | 2.6x10^7^ | N/a | 3.2x10^6^ |
| rYF/U-WT 1x10^4^ | 1x10^4^ | 2.6x10^6^ | N/a | 6.8x10^4^ |
| rYF/U- FVR 1x10^3^ | 1x10^3^ | 2.3x10^6^ | N/a | 3.2x10^4^ |
| rYF/U- FVR 1x10^2^ | 1x10^2^ | 2.3x10^5^ | N/a | 3.2x10^4^ |

* measured as a repeat experiment, so stocks had been sitting out for the day of infection, and then had a single defrost cycle. N/a – titres were not able to determined.

| **Experiment Three:**  **Group** | **Expected** | | **Per mouse** | |
| --- | --- | --- | --- | --- |
|  | **PFU/ml:** | **TCID50/ml:** | **PFU** | **TCID50** |
| rUSUV-WT 1x10^3^ | 1x10^3^ | 1.2x10^5^ | 7.8x10^2^ | 4.6x10^4^ |
| rYF/U-WT 1x10^3^ | 1x10^3^ | 2.6x10^5^ | 1.6x10^3^ | 6.8x10^4^ |
| rYF/U-WT 1x10^2^ | 1x10^2^ | 2.6x10^4^ | 2.3x10^2^ | 6.8x10^3^ |

##### Supplemental Table 3. Complete sequencing results of Bacmid (prelaunch) and passage 4 virus stock compared to reference sequences.

Key: Green highlight – Rationally Designed Mutation.

^*Mutations are present in sequencing results across different mutants.^

| a) rUSUV-FVR | Pre-Launch: | | Passage 4: | |
| --- | --- | --- | --- | --- |
| **Gene** | **Nucleotide Change** | **Amino Acid Change** | **Nucleotide Change** | **Amino Acid Change** |
| PrM | C576T (100%) | NA | C576T (99.7%) | NA |
| PrM |  |  | A616 + (5.1%)* | Insertion |
| PrM |  |  | T619 + (11.3%) | Insertion |
| PrM |  |  | G628 + (7.8%)* | Insertion |
| E | C1300T (100%) | E - L107F | T (99.6%) | E - L107F |
| E | A1302T (100%) |  | T (99.8%) |  |
| E | C1925T (100%) | E - A316V | T (99.2%) | E - A316V |
| E | T1926C (100%) |  | C (99.8%) |  |
| E | A2296C (100%) | E - K440R | C (99.9%) | E - K440R |
| E | A2297G (100%) |  | G (99.9%) |  |
| NS1 |  |  | C3449 + (5.3%)* | Insertion |

| b) rYF/U-WT | Pre-Launch: | | Passage 4: | |
| --- | --- | --- | --- | --- |
| **Gene** | **Nucleotide Change** | **Amino Acid Change** | **Nucleotide Change** | **Amino Acid Change** |
| C |  |  | C310A (5.4%) | C - H64Q |
| E |  |  | A1078T (5.1%) | E - I33F |
| NS5 |  |  | C9351 + A (7.9%) | Insertion |

| c) rYF/U- FVR | Pre-Launch: | | Passage 4: | |
| --- | --- | --- | --- | --- |
| **Gene** | **Nucleotide Change** | **Amino Acid Change** | **Nucleotide Change** | **Amino Acid Change** |
| PrM | C582T (100%) | NA | C582T (99.8%) | NA |
| PrM |  |  | G661A (12.3%) |  |
| PrM |  |  | A724C (12.5%) |  |
| M |  |  | T942G (8.6%)* | NA |
| M |  |  | C952G (5.8%)* | M - L66 |
| M |  |  | C958A (5.7%)* | M - L68I |
| E | C1300T (100%) | E - L107F | T (99.6%) | E - L107F |
| E | A1302T (100%) |  | T (99.8%) |  |
| E | C1925T (100%) | E - A316V | T (99.2%) | E - A316V |
| E | T1926C (100%) |  | C (99.8%) |  |
| E | A2296C (100%) | E - K440R | C (99.9%) | E - K440R |
| E | A2297G (100%) |  | G (99.9%) |  |
| NS1 |  |  | A3056G (5.3%) | NS1 K192R |
| NS1 |  |  | G3057A (5.9%) | NA |
| NS5 |  |  | C9351 + A (6.3%)* | Insertion |

##### Supplemental Table 4. Reference sequences used in sequence alignments for Figure 1.

| **Virus** | **Genbank Entry** |
| --- | --- |
| rUSUV-Af3 Recombinant Clone  - Strain TM Netherlands 2016 | PQ041659.1 |
| Usutu virus (USUV), complete cds  - NCBI Reference (Vienna 2001) | **NC_006551.1** |
| Murray Valley encephalitis virus (MVEV), complete genome.-  NCBI Reference Sequence | NC_000943 |
| Japanese encephalitis virus (JEV), genome  - NCBI Reference Sequence | NC_001437 |
| West Nile virus lineage 1, complete genome  - NCBI Reference Sequence | **NC_009942** |
| West Nile virus lineage 2, complete genome  -NCBI Reference Sequence | NC_001563 |
| Kunjin virus (KUNV) gene for polyprotein  -strain MRM61C | D00246.1 |
| Saint Louis encephalitis virus (SLEV), complete genome  -NCBI Reference Sequence | NC_007580 |
| Zika Virus (ZIKV), complete genome  -NCBI Reference Sequence | NC_012532.1 |
| Dengue virus 2 (DENV), complete genome  -NCBI Reference Sequence | NC_001474 |
| Yellow fever virus (YFV), complete genome  -NCBI Reference Sequence | NC_002031 |
